# Emergency management of fat embolism syndrome

**DOI:** 10.4103/0974-2700.44680

**Published:** 2009

**Authors:** Nissar Shaikh

**Affiliations:** Hamad Medical Corporation, P.Box 3050, Doha-Qatar

**Keywords:** Brain, clinical criteria, fat emboli, imaging studies, lung

## Abstract

Fat emboli occur in all patients with long-bone fractures, but only few patients develop systemic dysfunction, particularly the triad of skin, brain, and lung dysfunction known as the fat embolism syndrome (FES). Here we review the FES literature under different subheadings.

The incidence of FES varies from 1–29%. The etiology may be traumatic or, rarely, nontraumatic. Various factors increase the incidence of FES. Mechanical and biochemical theories have been proposed for the pathophysiology of FES. The clinical manifestations include respiratory and cerebral dysfunction and a petechial rash. Diagnosis of FES is difficult. The other causes for the above-mentioned organ dysfunction have to be excluded. The clinical criteria along with imaging studies help in diagnosis. FES can be detected early by continuous pulse oximetry in high-risk patients. Treatment of FES is essentially supportive. Medications, including steroids, heparin, alcohol, and dextran, have been found to be ineffective.

## INTRODUCTION

The fat embolism syndrome (FES) is a rare clinical condition in which circulating fat emboli or fat macroglobules lead to multisystem dysfunction.

In 1862, Zenker first described this syndrome at autopsy. In 1873, Von Bergmann clinically diagnosed FES for the first time.[1] Fat embolism occurs in all patients with long-bone fractures after intramedullary nailing. It is usually asymptomatic, but a few patients will develop signs and symptoms of multiorgan dysfunction, particularly involving the triad of lungs, brain, and skin.[2]

Here we review the FES literature under systematic subheadings.

## EPIDEMIOLOGY

The incidence of FES ranges from < 1 to 29% in different studies. It varies considerably according to the cause. The actual incidence of FES is not known, as mild cases often go unnoticed.

Bulger *et al*.,[3] in their retrospective study, reported an incidence of < 1%, while Fabian *et al*. in their prospective study, reported an incidence of 11–29%.[4] Surprisingly, the incidence was 0.9% when only clinical criteria were used to diagnose FES, whereas with the aid of postmortem examination the incidence was as high as 20%.[2]

## ETIOLOGY

FES is commonly associated with traumatic fracture of femur, pelvis, and tibia, and, postoperatively, after intramedullary nailing and pelvic and knee arthroplasty. The other forms of trauma that may be rarely responsible for FES include massive soft tissue injury, severe burn, bone marrow biopsy, bone marrow transplant, cardiopulmonary resuscitation, liposuction, and median sternotomy. The non-traumatic conditions are very uncommon causes of FES; they are acute pancreatitis, fatty liver, corticosteroid therapy, lymphography, fat emulsion infusion and haemoglobinopathies.[5]

## RISK FACTORS

The risk factors for the development of FES are young age, closed fractures, multiple fractures, and conservative therapy for long-bone fractures.[6] Factors which increase the risk of FES after intramedullary nailing are over-zealous nailing of the medullary cavity, reaming of the medullary cavity, increased velocity of reaming and increase in the gap between nail and cortical bone.

## PATHOPHYSIOLOGY

Two theories are postulated for the occurrence of FES. First, there is the mechanical theory by Gassling *et al*.,[7] which states that large fat droplets are released into the venous system; these droplets are deposited in the pulmonary capillary beds and travel through arteriovenous shunts to the brain. Microvascular lodging of the droplets produces local ischemia and inflammation, with concomitant release of inflammatory mediators and vasoactive amines and platelet aggregation. The biochemical theory states that hormonal changes caused by trauma and/or sepsis induce systemic release of free fatty acids as chylomicrons. Acute-phase reactants, such as C-reactive proteins, cause the chylomicrons to coalesce and create the physiologic reactions described above. Baker *et al*. blame the fatty acids for FES; the local hydrolysis of fat emboli by pneumocytes generates free fatty acids, which migrate to other organs via the systemic circulation, causing multiorgan dysfunction.[8] The biochemical theory helps in explaining the pathophysiology of the nontraumatic forms of FES. In an experimental study it is found that the intramedullary pressure increased up to 350 mm of Hg during reaming of the cavity.[9]

## CLINICAL FEATURES

The principal clinical features of FES are respiratory failure, cerebral dysfunction, and skin petechiae.

The clinical manifestations may develop 24–72 h after trauma (and especially after fractures) when fat droplets act as emboli, becoming impacted in the pulmonary microvasculature and other microvascular beds such as in the brain. Embolism begins rather slowly and attains a maximum in about 48 h.

The initial symptoms are probably caused by mechanical occlusion of multiple blood vessels with fat globules that are too large to pass through the capillaries. Unlike other embolic events, the vascular occlusion in fat embolism is often temporary or incomplete since the fat globules do not completely obstruct capillary blood flow because of their fluidity and deformability. The late presentation is thought to be a result of hydrolysis of the fat into the more irritating free fatty acids, which then migrate to other organs via the systemic circulation. It has also been suggested that paradoxical embolism occurs due to shunting.[10]

Pulmonary dysfunction is the earliest to manifest and is seen in 75% of patients; it progress to respiratory failure in 10% of the cases. The manifestations include tachypnea, dyspnea, and cyanosis; hypoxemia may be detected hours before the onset of respiratory complaints.[11] Cerebral changes are seen in 86% of patients with FES. These changes are nonspecific, ranging from acute confusion to drowsiness, rigidity, convulsions, or coma. Cerebral edema contributes to the neurological deterioration.[12]

The skin dysfunction is manifested as a nonpalpable petechial rash in the chest, axilla, conjunctiva, and neck that appears within 24–36 h and disappears within a week in 20–50% of patients. The particular distribution of the rash is related to the fact that the fat particles float in the aortic arch like oil in water and thus get embolized to the nondependent areas of the body.[13]

Several other signs are nonspecific, like tachycardia and pyrexia. Renal changes may include lipuria, oliguria, or anuria and hepatic damage may manifest as jaundice. The retina may show exudates, edema, hemorrhage, or intravascular fat globules.[14]

There may be history of orthopedic or plastic surgical procedure or parenteral lipid transfusion.

## DIFFERENTIAL DIAGNOSIS

Dyspnea and hypoxia can also occur with pulmonary embolis and pneumonia. Cerebral dysfunction will occur with hypoxia or meningitis, but the rash of meningococcal septicemia spreads rapidly all over the body.

## DIAGNOSIS

FES is commonly diagnosed on the basis of the clinical features and by excluding other causes. Gurd's and Wilson's criteria[11] are shown in [Table 1]; diagnosis of FES requires the presence of at least one major criteria and at least four minor criteria.

**Table 1:** Gurd's and Wilson's criteria

Major criteria
Petechial rash
Respiratory insufficiency
Cerebral involvement

Minor criteria
Tachycardia
Fever
Retinal changes
Jaundice
Renal signs
Thrombocytopenia
Anemia
High ESR
Fat macroglobinemia

Schonfeld *et al*. proposed [Table 2] a quantitative measure to diagnose FES; a score of more than 5 is required to diagnose FES.[15]

**Table 2:** Schonfeld's criteria

	Score
Petechiae	5
X-ray chest diffuse infiltrates	4
Hypoxemia	3
Fever	1
Tachycardia	1
Tachypnea	1
Confusion	1

According to Lindeque *et al*., [Table 3] FES can be diagnosed on the basis of respiratory system involvement alone.[16]

**Table 3:** Lindeque's criteria

Sustained pO_2_ < 8 kpa
Sustained pCO_2_ > 7.3 kpa
Sustained respiratory rate >35/min, in spite of sedation
Increased work of breathing, dyspnea, tachycardia, anxiety

### Investigations

Arterial blood gas analysis showing an unexplained increase in pulmonary shunt fraction and an alveolar-to-arterial oxygen tension difference, especially within 24–48 h of a sentinel event associated with FES, is strongly suggestive of the diagnosis. Blood gases will show hypoxia, with a paO_2_ of less than 60 mmHg along with the, and presence of hypocapnia.

Thrombocytopenia, anemia, hypofibrinogenemia, and increased erythrocyte sedimentation rate (ESR) are seen in FES, but are nonspecific findings. A decrease in hematocrit occurs within 24–48 h and is attributed to intra-alveolar hemorrhage.

Cytological examination of urine, blood, and sputum may detect fat globules that are either free or within macrophages. This test is not sensitive and its absence does not rule out fat embolism. Fat globules in the urine are common after trauma. Preliminary investigations of the cytology of pulmonary capillary blood obtained from a wedged pulmonary artery catheter revealed fat globules in patients with FES and showed that this method may be beneficial in early detection of patients at risk.[17]

### Imaging studies

**Figure 1:**
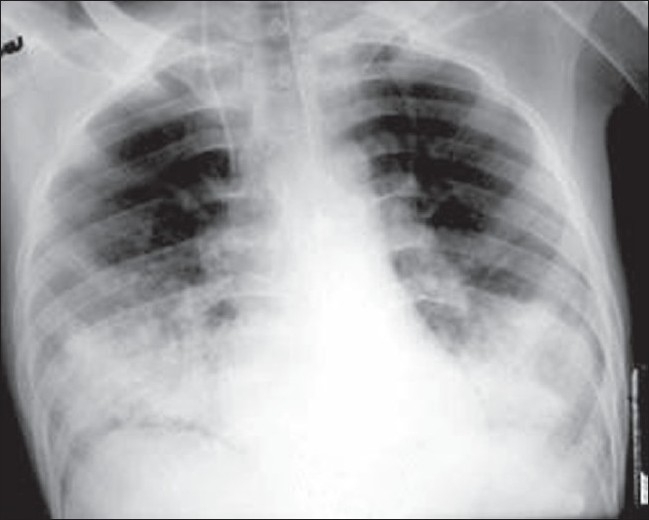
AP radiograph of the chest showing bilateral basal air space–filling lesions (consolidation)

Chest radiography: Serial radiographs reveal increasing diffuse bilateral pulmonary infiltrates [Figure 1], fleck-like pulmonary shadows (‘snow storm’ appearance), increased pulmonary markings, and dilatation of the right side of the heart within 24–48 h of onset of clinical findings.

CT (computerized tomography) head: Findings may be normal or may reveal diffuse white-matter petechial hemorrhages consistent with microvascular injury. CT will also rule out other causes for deterioration in consciousness level.

Ventilation/perfusion imaging of the lungs: Performed for suspicion of pulmonary embolus, the findings from this scan may be normal or may demonstrate subsegmental perfusion defects.

Spiral chest CT for pulmonary embolism: As the embolic particles are lodged in the capillary beds, findings of spiral chest CT may be normal. Parenchymal changes consistent with lung contusion, acute lung injury, or adult respiratory distress syndrome (ARDS) may be evident.[17]

Magnetic resonance imaging (MRI) brain: Scanty data exist regarding MRI findings in patients with this syndrome; however, in one small patient group, multiple, nonconfluent, hyperdense lesions were seen on proton-density and T2-weighted images.[18]

**Figure 2:**
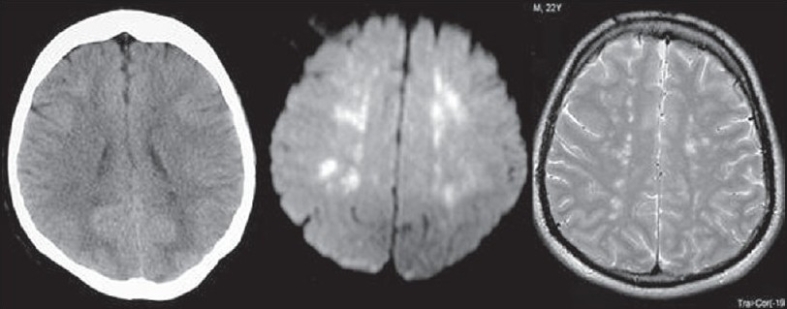
CT image showing minimal hypodense changes in periventricular region, which are more evident in DWI and T2WI as areas of high signals. Constellation of findings along with clinical data is characteristic for FES.

Magnetic resonance imaging brain is more sensitive than CT scan and diagnosis can be made earlier. It will show typical white matter changes along the boundary zones of major vascular territories [Figure 2].[19]

Transcranial Doppler sonography: In a small case study, five patients with trauma were monitored with intracranial Doppler sonography during intraoperative nailing of long-bone fractures. Cerebral microembolic signals were detected as late as 4 days after injury.[20]

Transesophageal echocardiography (TEE): This procedure may be of use in evaluating intraoperative release of marrow contents into the bloodstream during intramedullary reaming and nailing. The density of the echogenic material passing through the right side of the heart correlates with the degree of reduction in arterial oxygen saturation. Repeated showers of emboli have been noted to increase right heart and pulmonary artery pressures. Embolization of marrow contents through a patent foramen ovale has also been noted.[21]

### Procedures

Bronchoalveolar Lavage (BAL) staining of alveolar macrophages for fat will demonstrate fat droplets thus enabling a rapid and specific diagnosis of FES.[22] But one has to be careful, as fat droplets in BAL may be present in patients with sepsis and hyperlipidemia. They may also be seen in patients on lipid infusions. Presently, the use of BAL to aid in the diagnosis or to predict the likelihood of FES is controversial.[23]

## TREATMENT

Treatment of FES consists of ensuring good arterial oxygenation. High flow rate oxygen is given to maintain the arterial oxygen tension in the normal range. Additionally, maintenance of intravascular volume is important, because shock can exacerbate the lung injury caused by FES. Albumin has been recommended for volume resuscitation in addition to balanced electrolyte solution, because it not only restores blood volume but also binds with the fatty acids and may thus decrease the extent of lung injury. Mechanical ventilation and PEEP may be required to maintain arterial oxygenation.[24] Medications, including steroids, heparin, alcohol, and dextran, have been found to be ineffective.[25]

## PREVENTION

Continuous pulse oximetry monitoring in high-risk patients may help in detecting desaturation early, allowing early institution of oxygen (and possibly steroid) therapy; it would thus be possible to decrease the chances of hypoxic insult and the systemic complications of FES.[26] The early fixation of long-bone fracture is important to prevent or to decrease the severity of FES.[27] External fixation or fixation with plate and screw produces lesser lung injury than nailing the medullary cavity and venting the medullary canal during nailing, reduces the number of emboli.[28] Preoperative use of methylprednisolone may prevent the occurrence of FES.[15] Smaller-diameter nails and unreamed nailing have been mentioned as being useful in the prevention of FES.

## PROGNOSIS

The fulminant form presents as acute cor pulmonale, respiratory failure, and/or embolic phenomena, leading to death within a few hours of injury. Patients with increased age, multiple underlying medical problems, and/or decreased physiologic reserves have worse outcomes.[29]

The duration of FES is difficult to predict because FES is often subclinical or overshadowed by other illnesses or injuries. Increased alveolar-to-arterial oxygen gradient and neurology deficits, including altered consciousness, may last days or weeks. As in ARDS, the pulmonary sequelae usually resolve almost completely within a year. Residual subclinical diffusion capacity deficits may persist.

Residual neurological deficits may range from subtle personality changes to memory loss, cognitive dysfunction and long term focal deficits. FES alone has not yet been reported to cause global anoxic injury, but it may play a contributory role, acting along with other cerebral insults. The mortality rate from FES is 5–15%. Even severe respiratory failure associated with fat embolism seldom leads to death.

## CONCLUSION

A high index of suspicion is needed to diagnose FES. A combination of clinical criteria and MRI brain will enable early and accurate diagnosis of FES.
